# TLR9 activation via microglial glucocorticoid receptors contributes to degeneration of midbrain dopamine neurons

**DOI:** 10.1038/s41467-018-04569-y

**Published:** 2018-06-22

**Authors:** Layal Maatouk, Anne-Claire Compagnion, Maria-Angeles Carrillo-de Sauvage, Alexis-Pierre Bemelmans, Sabrina Leclere-Turbant, Vincent Cirotteau, Mira Tohme, Allen Beke, Michaël Trichet, Virginie Bazin, Bobby N. Trawick, Richard M. Ransohoff, François Tronche, Bénédicte Manoury, Sheela Vyas

**Affiliations:** 1 Institute of Biology Paris Seine, Gene Regulation and Adaptive Behaviors Team, Department of Neuroscience Paris Seine, Sorbonne Université, CNRS UMR 8246 & INSERM U1130, 9 Quai Saint Bernard, F-75005 Paris, France; 2 CEA, DRF, Institut François Jacob, Neurodegenerative Diseases Laboratory, Molecular Imaging Research Center (MIRCen), CNRS, CEA, Université Paris-Sud, Université Paris-Saclay (UMR9199), F-92265 Fontenay-aux-Roses, France; 3 IHU-A-ICM–Neuro-CEB, Plateforme de Ressources Biologiques (PRB), Hôpital de la Pitié-Salpétrière, 47 Boulevard de l’Hôpital, F-75013 Paris, France; 4 INEM, INSERM U1151-CNRS UMR 8253, Hôpital Necker, Université Paris Descartes, Sorbonne Paris Cité, Faculté de Médecine, 149 Rue de Sèvres, F-75005 Paris, France; 5 Institute of Biology Paris Seine, Electron Microscopy Facility, Sorbonne Université FR3631, 9 Quai Saint Bernard, F-75005 Paris, France; 6 Center for Organic Chemistry, Mallinckrodt Pharmaceuticals, 3600N. Second Street, B81-T l, St. Louis, MO 63147 USA; 7 Third Rock Ventures, Boston, MA 02116 USA

## Abstract

Inflammation is a characteristic feature of Parkinson’s disease (PD). We examined the role of TLR9 and its regulation by glucocorticoid receptors (GRs) in degeneration of substantia nigra dopamine neurons (DNs). TLR9 agonist, CpG-ODN, induced DN degeneration in mice lacking GR in microglia but not in controls. TLR9 deletion reduced DN loss in neurotoxin, 1-methyl-4-phenyl-1,2,3,6-tetrahydropyridine (MPTP) mouse model of PD. GR regulates TLR9 activation during MPTP neurotoxicity as TLR9 antagonist suppressed increased DN loss in microglia/macrophage GR mutant mice. GR absence in microglia enhanced TLR9 translocation to endolysosomes and facilitated its cleavage leading to pro-inflammatory gene expression. GR-dependent TLR9 activation also triggered DN loss following intranigral injection of mitochondrial DNA. Finally, microglial GR sensitivity to A53T-alpha-synuclein induced DN degeneration as well as decreased microglial GR expression observed in SN of PD brain samples, all suggest that reduced microglial GR activity in SN can stimulate TLR9 activation and DN loss in PD pathology.

## Abstract (web summary)

Inflammation is a component of Parkinson’s disease. Here the authors show that activation of Toll-like receptor 9 controlled by microglial glucocorticoid receptor signaling, contributes to dopamine neuron loss in a model of Parkinson’s disease.

## Introduction

Microglial reactivity is an early and characteristic feature of Parkinson’s disease (PD). Increasing evidence both from clinical and animal studies point to active microglial involvement, through secretion of inflammatory mediators, in the development and progression of PD pathology^1–3^. Progressive degeneration of dopamine neurons (DNs) in substantia nigra pars compacta is a hallmark of PD pathology responsible for motor symptoms^4^. Among brain regions, SN has a high density of microglia^5^, and work with experimental PD models indicates a selective vulnerability of DNs to inflammatory attack by reactive microglia^6^.

Microglia are the principle resident innate immune cells of the brain expressing diverse classes of pattern recognition receptors including all TLR (Toll-like receptors) family members^7^. TLRs are activated by both PAMPs (pathogen-associated molecular patterns) present in microbes and DAMPs (danger-associated molecular patterns) present in molecules released by damaged cells, which leads to an intracellular signaling cascade and transcription-dependent inflammatory gene expression^8,9^. NF-κB, AP-1, and IRF are major transcriptional factors involved in orchestrating the innate immune responses upon TLR ligand binding^10^. The functional roles of NF-κB and AP-1 in microglial-mediated inflammatory response and death of DNs have been demonstrated in experimental PD, while the presence of p65 subunit of NF-κB or JNK in microglia of SN was shown in PD post-mortem studies^11,12^. Several lines of evidence, based on animal studies, reinforce the idea that TLRs play a role in degeneration of DNs. Intranigral injection of LPS, through TLR4 activation in microglia, induces specific loss of DNs^13^. Similarly, intranigral injection of poly I:C, activating TLR3 in microglia, was found to lower the threshold of vulnerability of DNs for the neurotoxin 6-OHDA^14^. Neuronally released oligomeric forms of α-synuclein were shown to activate microglial TLR2^15^, whereas we previously showed that MPTP (1-methly-4-phenyl-1,2,3,6-tetrahydropyridine) intoxication in mice significantly upregulates TLR9 as well as TLR 4, 7 and the key TLR adapter protein MyD88^16^.

Although microglial-mediated inflammatory response can exacerbate or trigger DN cell death, regulatory mechanisms in these cells normally limit innate immune actions. Among the factors controlling inflammatory reaction, the nuclear receptors such as GR, PPAR-γ, LXR, or Nurr1 represent an important group shown to protect DNs in experimental Parkinsonism through regulation of transcriptional activities of NF-κB and AP-1 in microglia^16–19^. In our previous work on the regulatory role of GR in microglia during degeneration of DNs triggered by the neurotoxin MPTP, we found increased levels of TLR3, 4, and 9 in SN of mice lacking GR in microglia/macrophages (GR^LysMCre^ mutant mice) when compared to controls^16^, suggesting a role of GR in controlling their expression. Interestingly, in the same study, a significant upregulation of TLR9 protein in the striatum of PD post-mortem brains compared to age-matched control subjects was also observed, pointing to a putative role of TLR9 in PD pathogenesis.

In this study, first we provide further evidence for the role of microglial GR in PD pathogenesis. In the absence of GR in microglia, there is increased death of DNs following intranigral injection of AAV-A53T-α synuclein. The number of microglia-expressing GR is significantly reduced in the SN of post-mortem PD compared to control brain tissue suggesting that this loss of GR in microglia could contribute to dopamine neurodegenerative process. We then show the tight regulatory role of microglial GR in TLR9 activation. This has direct consequences for the loss of DNs in SN triggered either by TLR9 agonists or by MPTP. Overall, these results indicate that reduced GR activity in microglia sensitizes microglia to TLR9 activation, which is detrimental to survival of DNs in PD pathogenesis.

## Results

### The role of microglial GR in PD pathology

**Fig. 1:**
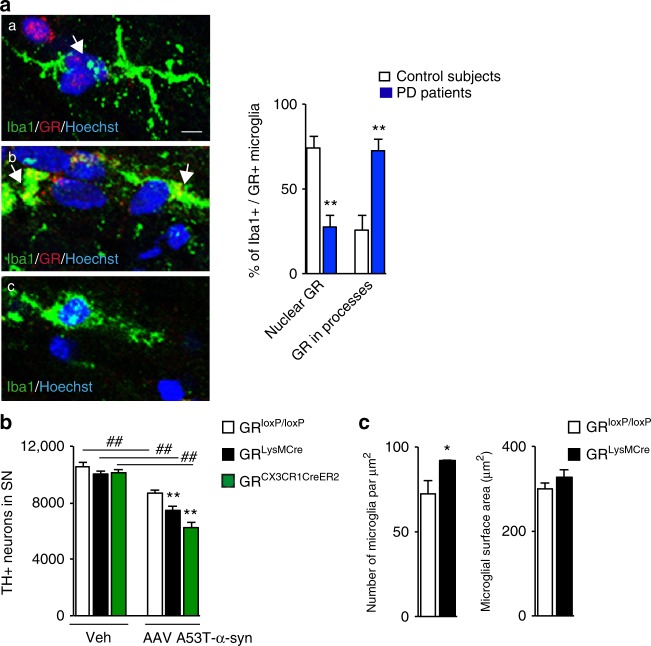
Involvement of microglial GR in PD pathology. **a** Left: Representative confocal micrographs of Iba1 staining of microglia in SN of human post-mortem sections and GR presence: **a** in the nucleus (shown by arrows); **b** in microglia processes (shown by arrows); **c** absence of GR. Quantification of GR in Iba1 + microglia and GR co-localization in sections from control subjects and PD patients. Bar = 5 μm. The results are presented as % of GR + microglia quantified from sections of each individual; *n* = 4 for control subjects and PD patients. **p ≤ 0.02 control subjects vs. PD patients. **b** TH-IR neurons in SN of GR^loxP/loxP^, GR^LysMCre^, and GR^LysMCre^ mice quantified 7 days after a single intranigral injection of vehicle (saline) or AAV-PGK-A53T α-synuclein. *n* = 3–4 in saline group and *n* = 5–7 for AAV-PGK-α-synuclein group, ##*p* ≤ 0.02; vehicle saline vs. AAV-PGK-A53Tα-synuclein injection, **p ≤ 0.02 GR^loxP/loxP^ vs. GR^LysMCre^ or GR^CX3CR1CreER2^ mice after AAV-PGK-A53Tα-synuclein injection. **c** Quantification of microglial density and surface area in same GR^loxP/loxP^ and GR^LysMCre^ mice after AAV-PGK-A53T□α-synuclein injection. *p < 0.05, GR^loxP/loxP^ vs. GR^LysMCre^ mice. The data presented as mean and error bars indicate s.e.m. Statistical significance determined in all by Mann–Whitney non-parametric test

The transcriptional activation of GR is mediated by binding of glucocorticoids (GCs) to inert GR–protein complex in the cytoplasm provoking GR translocation to nucleus. To examine whether microglial GR expression is altered during dopamine neurodegeneration in PD, we analyzed its localization in microglia in SN post-mortem brain sections from age and sex-matched control subjects and PD patients (*n* = 4). Double immunofluorescence (IF) labeling of Iba1 and GR showed GR presence in the nucleus, in processes and microglia without GR (Fig. 1a). GR expression was quantified and the result, expressed as % total GR + Iba + microglia shows % nuclear GR localization almost reversed in PD compared to controls. Thus 74 ± 7.2% (mean ± s.e.m.) of GR + Iba + microglia in controls display nuclear localization, which is reduced to 27.5 ± 6.8% in PD (*p* = 0.02), with parallel increase of GR in processes, suggesting a dysfunction of GR signaling in PD.

Alpha-synuclein is intimately involved in PD pathological processes that lead to degeneration of DNs in SN. Recently, AAV A53T-α-synuclein injection in SN was shown to trigger degeneration of DNs^20^. To examine whether microglial GR is involved in regulating DN degeneration triggered by pathological A53T-α-synuclein, we injected AAV-PGK-A53T-α-synuclein in intranigral region of control mice and two microglial GR mutant lines: GR^LysMCre^ mice in which GR gene is not only inactivated in microglia but also in peripheral myeloid cells^16,21,22^ and GR^CX3CR1CreER^ where long-term gene inactivation occurs specifically in microglia^23^. The absence of GR in microglial cells in GR^LysMCre^ mice has been verified previously^16,21^. We verified the absence of GR in GR^CX3CR1CreER2^ after tamoxifen injections in control and mutant mice (Supplementary Fig. 1A) and find around 80% of microglia have no GR labeling compared to controls (Supplementary Fig. 1B).

Eight weeks following adenoviral A53T-α-synuclein injection, the mice were sacrificed and the number of tyrosine hydroxylase immunoreactive (TH-IR) neurons quantified in SN. A decrease in the number of TH-IR neurons was observed in all three genotypes (*p* = 0.02, 0.017, and 0.03 for control, GR^LysMCre^ and GR^CX3CR1CreER2^ mutants; vehicle vs. A53T-α-synuclein virus injection). Importantly, there was greater decrease in GR^LysMcre^ and GR^CX3CR1CreER2^ mice compared to control GR^loxP/loxP^ mice (*p* = 0.01 and 0.02, respectively, for mutants compared to control mice) (Fig. 1b). A higher density of microglia indicative of increased proliferation was seen in GR^LysMCre^ mutant mice compared to controls (Fig. 1c). Thus, GR in microglia acts to prevent degeneration of DNs induced by pathological form of α-synuclein.

### TLR9 triggered DN loss in SN is controlled by microglial GR

**Fig. 2:**
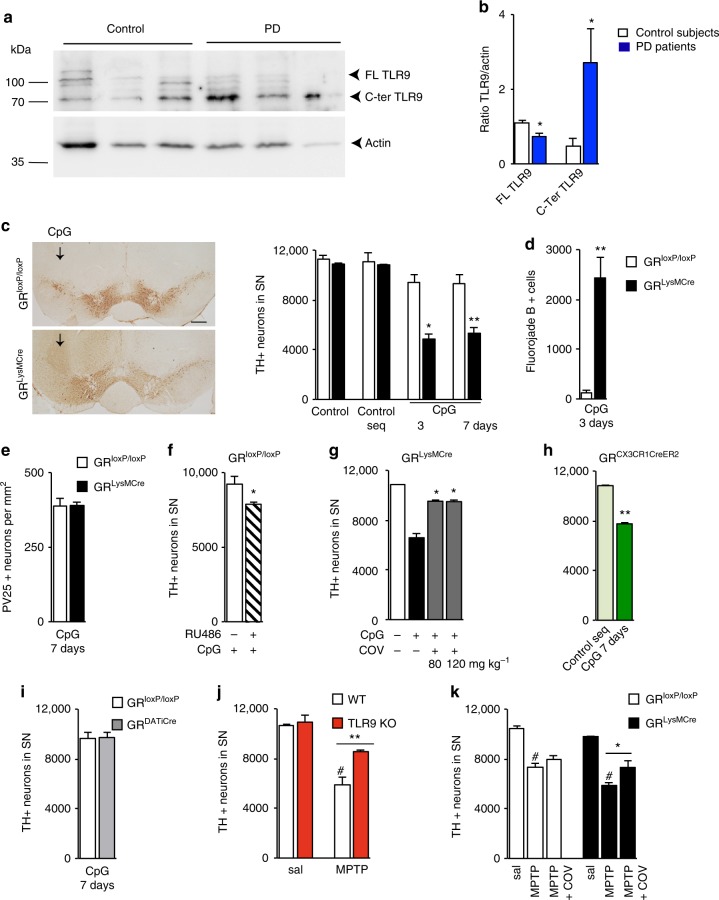
Microglial GR regulates TLR9 signaling which affects DN survival in substantia nigra. **a** WB of TLR9 and actin in total SN homogenates from control subjects and PD patients. **b** Quantification of full-length (FL) and C-terminal fragment (C-ter) *p < 0.05, PD vs. control, *n* = 3. Mann–Whitney non-parametric test. Full gel blots in Supplementary Fig. 4. **c** TH immunohistochemistry in SN of GR^LysMCre^ and GR^loxP/loxP^ mice 7 days after a single intranigral injection CpG ODN showing loss of DNs in mutants. Bar = 100 μm. Total number of TH-IR DNs spanning entire SN were quantified 3 and 7 days after intranigral injection of saline (sal), control sequence (c-seq), or CpG ODN. *n* = 5 per group. *p < 0.05, **p ≤ 0.02, control vs. mutant, post hoc Bonferroni/Dunn test. **d** Degenerating fluorojade-positive neurons in SN of controls and GR^LysMCre^ mice were quantified 3 days after a single intranigral injection of CpG ODN. **p ≤ 0.02; controls vs. mutants, Mann–Whitney test, *n* = 5 per group. **e** Quantification of parvalbumine-positive GABAergic neurons in SN of controls and GR^LysMCre^ mice 7 days after a single intranigral injection of CpG ODN. **f** TH-IR neurons in SN of GR^loxP/loxP^ mice, pretreated or not with i.p. injection of GR antagonist RU486 quantified in SN 7 days after a single intranigral injection of CpG ODN *p < 0.05; pretreated with RU 486 vs. non-pretreated, Mann–Whitney test, *n* = 5/group. **g** TH-IR neurons in SN after injection of either 80 or 120 mg kg^−1^ COV08-0064 1 h i.p. prior to stereotaxic injection of CpG ODN in GR^LysMCre^ mice, and x1 for 3 days, mice sacrificed after 3 days. *n* = 4/group *p < 0.05 pretreated with COV08-0064 vs. CpG ODN only. Mann–Whitney test. **h** Quantification of TH-IR cells in GR^CX3CR1CreER2^ mice injected with control sequence (c-seq) or CpG ODN. **p ≤ 0.02 control seq vs. CpG ODN, Mann–Whitney test *n* = 5 group. **i** Quantification of TH-IR cells in SN in control and GR^DATiCre^ mice after CpG ODN injection. *n* = 4/group. **j** Saline or MPTP injections (20 mg kg^−1^) in wild-type (WT-C57BL/6) or TLR9 k.o. mice followed by quantification of TH-IR cells in SN. *n* = 3/saline group and 4–5 for MPTP group. #*p* < 0.05 saline vs. MPTP, **p ≤ 0.02 WT MPTP vs. TLR9 k.o MPTP, Mann–Whitney test. **k** Quantification of TH-IR cells in SN 7 days after saline (sal), MPTP (18 mg kg^−1^), or MPTP + COV08-0064 treatment in GR^loxP/loxP^ and GR^LysMCre^ mice. *n* = 3/saline group, *n* = 5/MPTP group, *n* = 5/MPTP + COV08-0064 control group, and *n* = 3/MPTP + COV08-0064 mutant group. #*p* < 0.05 saline vs. MPTP and **p ≤ 0.02 MPTP vs. MPTP + COV08-0064 group, Mann–Whitney test. All data are presented as mean and error bars indicate s.e.m

In light of our previous results showing high TLR9 protein levels in the post-mortem striatum of PD^16^, as well as regulation of inflammatory gene transcription by GR upon TLR9 pathway stimulation^24^, we also examined TLR9 levels in SN of PD. Western blot results of TLR9 protein levels and quantification with respect to actin levels in post-mortem SN tissue from three PD patients and three age-matched controls showed a decrease in full-length (FL) TLR9 protein levels (Fig. 2a, b). A second band whose size corresponds to C-terminal active form of TLR9^25^ was observed (Fig. 2a). Quantification revealed an increase in C-terminal fragment in SN of PD suggesting a likelihood of TLR9 activation in SN during pathogenesis of PD (Fig. 2b).

The role of microglial GR in TLR9-inflammatory-mediated degeneration of DNs was next examined in control, GR^LysMCre^, and GR^CX3CR1CreER2^ mice. TLR9 sensing of stretches of DNA containing the cytosine-phosphate-guanosine oligodeoxynucleotide (CpG ODN) motif present in bacteria is well characterized^26^. Control and mutant GR^LysMcre^ mice (*n* = 5 for each time point and genotype) were injected unilaterally with CpG ODN or control ODN sequence (each 20 bp long, 0.96 μg μl^−1^) in the intranigral region. The mice were sacrificed either at 3 or 7 days after injections and total number of TH-IR DNs quantified. Injections of control ODN sequence in control or in mutant GR^LysMCre^ mice had no effect on DN survival when compared to saline-injected mice (Fig. 2c). In control GR^loxP/loxP^ mice, there was no significant decrease in the number of DNs following CpG ODN injection (*p* = 0.48 for 3 days and 0.09 for 7 days, CpG ODN vs. control ODN). In contrast, in GR^LysMcre^ mutant mice, the same dose of CpG ODN triggered significant loss of DNs at both time points of 3 and 7 days (*p* < 0.02 control vs. mutant, post hoc Bonferroni/Dunn test) (Fig. 2c). To ensure that the decrease in the number of DNs is not reflecting disappearance of TH protein, sections were stained for Fluoro-Jade B (which labels degenerating neurons) 3 days after intranigral injection of CpG ODN. Fluoro-Jade B-positive cells were seen in the supra-nigral region, i.e., along the track of the injection (Supplementary Fig. 2A) as well as in SN (Supplementary Fig. 2B, C). Quantification of Fluoro-Jade B-positive cells in SN of control and mutant mice showed increase in degenerating neurons in GR microglial/macrophagic mutants compared to control mice (*p* < 0.003) (Fig. 2d). The parvalbumin immunoreactive neurons were quantified in SN region after CpG ODN injection both in control and GR microglial/macrophage mutant mice to evaluate whether neurodegeneration was selective with regard to DNs. The results showed the same number of neurons in control and mutant mice (Fig. 2e). We also verified whether acute global pharmacological GR inhibition affects DN loss. Control mice were i.p. injected with vehicle or GR antagonist RU486 (30 mg kg^−1^) 16 h prior to CpG ODN intranigral injection. The loss of DNs was observed (*p* = 0.05) in RU486-injected mice compared to control vehicle-pretreated mice (Fig. 2f). To verify the involvement of TLR9 in CpG ODN-induced DN death we tested TLR9 antagonist COV08-0064. The specificity and actions of COV08-0064 were reported in the peripheral tissues^27,28^ and not in CNS. However, it most likely traverses the blood brain barrier as indicated by its physico-chemical properties and the calcein-AM analysis in MDR1-MDCKII cell line showing it is not a substrate for CNS Pgp efflux transporter (Supplementary Note 1, Supplementary Tables 1 and 2). Mutant GR^LysMcre^ mice were i.p. injected with 80^28^ and 120 mg kg^−1^ TLR9 antagonist COV08-0064, 1 h prior to CpG ODN intranigral injection as well as once per day until sacrifice after 3 days. There was significant reversal of CpG ODN effect in the presence of COV08-0064 (*p* = 0.03) indicating that COV08-0064 is antagonizing TLR9, plausibly both in macrophages and in microglia in SN as GR is inactivated in myeloid cells in GR^LysMcre^ mice (Fig. 2g).

To show the involvement of GR only in microglia, CpG ODN was injected in GR^CX3CR1CreER2^ mice. In these mice, there was significant (*p* = 0.01) decrease in the number of TH + neurons in SN after CpG ODN injection compared to injection of control sequence (Fig. 2h). Thus, TLR9 activation by synthetic ligand, CpG ODN, triggers significant DN loss in the absence of microglial GR activity.

DNs were reported to express TLR3 and injection of its ligand poly I:C was shown to render these neurons susceptible to 6-OHDA^29^. Since the above results show that DN loss by CpG ODN is dependent on GR, we looked for any direct effect of CpG ODN on DNs by injecting CpG ODN in the intranigral region of mice inactivated for GR gene specifically in DNs (GR^DATiCre^). Of note the absence of GR in DNs has been verified and reported previously^30^. Comparable numbers of DNs were found in in GR^DATiCre^ mutant and GR^loxP/loxP^ control mice following CpG injection (Fig. 2i) suggesting that GR in DNs does not play a role in TLR9- induced toxicity.

Previously, we showed upregulation of TLR9 during DN death triggered by MPTP^16^. To show that endogenous TLR9 plays a role in MPTP-triggered DN death, TLR9 gene targeted and WT mice were injected x4 with 20 mg kg^−1^ MPTP at 2 h interval, a paradigm known to activate glia^31^. Absence of TLR9 protected DNs against MPTP (*p* = 0.01) (Fig. 2j), suggesting that TLR9 is activated during dopamine neurodegeneration triggered by MPTP. To show the involvement of GR in restraining TLR9 activation during MPTP-induced dopamine neurodegeneration, control and GR^LysMcre^ mutant mice were injected with saline, MPTP, and with COV08-0064 prior to MPTP injection and once for 2 days consecutive following MPTP injections. The results show increased viability of DNs after COV08-006 treatment in GR^LysMcre^ mice compared to controls (*p* = 0.03) (Fig. 2k).

### Microglial GR does not affect DN loss triggered by TLR4 and TLR7

To determine whether the regulation of DN loss by microglial GR also extends to other members of TLR family, we examined the effects of TLR2, TLR4, and TLR7 activation. We performed stereotaxic injections of the TLR4 agonist, LPS (0.5, 1, and 1.5 μg μl^−1^), TLR2 agonist, pam3cys (1 μg μl^−1^), and TLR7 agonist, imiquimod (1 μg μl^−1^) in the SN region of control GR^loxP/loxP^ and mutant GR^LysMcre^ mice. The mice were sacrificed 3 and/or 7 days after the injection. With LPS at 0.5 μg μl^−1^, a tendency for a decrease was observed whereas there was an inter-individual variability at 1 μg μl^−1^ in the GR^LysMcre^ mice (Supplementary Fig. 3A). As expected and previously reported^13^, LPS induced significant DN degeneration at 1.5 μg μl^−1^ in control mice; the absence of GR in microglia did not trigger further degeneration in mutant mice (*p* < 0.03) (Supplementary Fig. 3A). The number of DNs was similar after Pam3cys injection in control and mutant mice compared to saline-injected mice, indicating that TLR2 stimulation by Pam3cys has no effect on DN survival (Supplementary Fig. 3B). On the other hand, stimulation of TLR7 by imiquimod triggered DN degeneration, which was similar in control and mutant mice (*p* = 0.03) (Supplementary Fig. 3C). These results suggest that among the TLR family members tested, only TLR9 activation is sensitive to microglial GR in SN.

### Deficit in motor performance following TLR9-induced DN loss

**Fig. 3:**
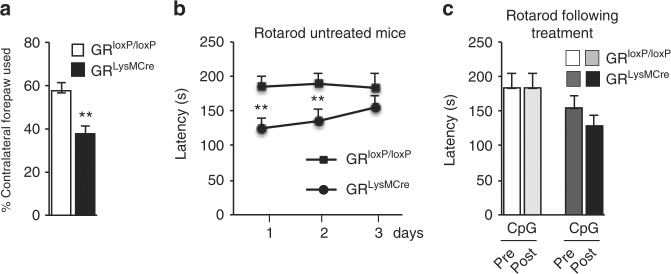
Motor behavior tests in control and GR^LysMcre^ mice before and after unilateral intranigral CpG-ODN injection. **a** Analysis of cylinder test: % of contralateral forelimb touch onto cylinder surface in an upright position out of 20 total ipsilateral and contralateral touches after unilateral CpG-ODN injection. **p < 0.01 control vs. mutant. **b** After habituation on rotarod, the control and mutant mice were scored for latency to fall on accelerating rotarod. **p < 0.01 control vs. mutant. **c** Mice retested on accelerating rotarod 1 week after CpG-ODN intranigral injection. The control mice at day 3 showed same latency as during preinjection testing whereas there was a small non-significant decrease in latency with mutant mice. *n* = 9 for each group. All data are mean of three trials with error bars as s.e.m., with Mann–Whitney non-parametric test for statistical significance

As a correlate of DN loss in SN, tests for any motor deficits were undertaken. Since our lesion was unilateral we used cylinder test as a measure of forelimb akinesia^32^ in control and mutant mice 12 days following CpG-ODN injection. The results showed significant reduction (*p* = 0.009) (approximately 35%) in the use of contralateral paw onto surface of cylinder in mutant compared to control mice (Fig. 3a). We also used accelerating rotarod paradigm to test both for motor performance and learning in control and GR^LysMcre^ mutant mice just before and 1 week after intranigral CpG ODN injection. After habituating all mice to stay on accelerating rotarod (5–40 r.p.m.) for maximum period (3–5 min), latency to fall on accelerating rotarod was recorded 1 week later, 3 trials/day for 3 days. The results show that over 3 days, control mice show same latency to fall, indicative of learned motor performance. However, GR^LysMcre^ mutant mice have impaired learned motor behavior as their latency is significantly below controls at day 1 and gradually increases attaining almost same latency as controls on day 3 (Fig. 3b). The mice were re-tested 1 week after intranigral CpG-ODN injection. Similar difference in latency to fall as in preinjection testing was observed between control and mutant mice at day 1 and 2 (Fig. 3c). In this regard, DN loss in SN is not always perceived by latency to fall in rotarod rod testing^33,34^.

### GR-dependent TLR9 activation triggers pro-inflammatory state

**Fig. 4:**
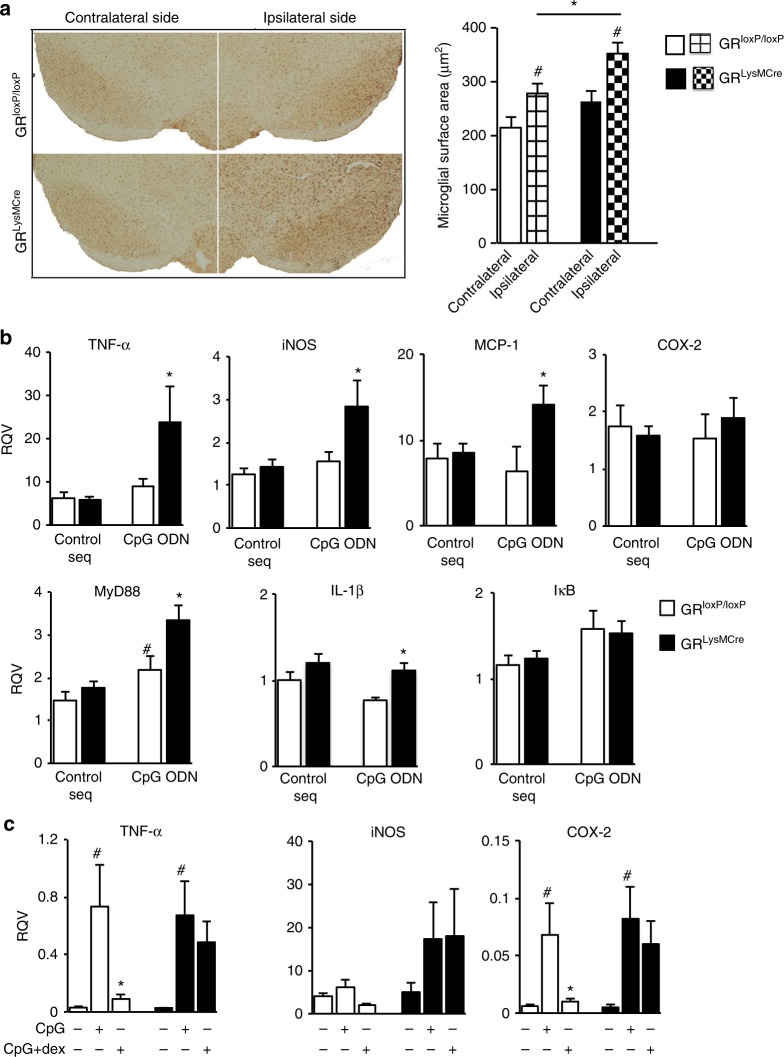
Microglial GR regulates microglial reactivity and pro-inflammatory gene expression after CpG ODN injection. **a** Immunohistochemistry of Iba-1 7 days following CpG ODN injection both in controls and mutant mice. Quantification of microglial surface area in SN on contra-lateral and ipsi-lateral sides of GR^LysMCre^ mutant and control mice a.u: arbitrary units. *p < 0.05 control vs. mutant; #*p* < 0.05 ipsilateral vs. contralateral side. *n* = 5/group. **b** In vivo analysis of inflammatory gene levels by RT-qPCR 24 h after CpG ODN injection in control and GR^LysMCre^ mice. The RQV (relative quantitative value) was calculated using HPRT gene as internal control and compared to contralateral non-injected side. Increased levels are observed in mutants compared with controls at 24-h time point. *p < 0.05, control vs. GR^LysMCre^ mutant CpG-injected mice, *n* = 4–5. **c** In vitro analysis of pro-inflammatory gene levels by RT-qPCR in control and mutant microglial cultures 40 min after control sequence (c-seq), CpG, or CpG + dexamethasone (dex) treatment. The RQV was calculated using beta-microglobulin as internal control. The data are from three independent experiments. #*p* < 0.05 c-seq vs. CpG, *p < 0.05 CpG vs. CpG + dex. All data presented are mean and error bars indicate s.e.m. The statistical significance determined by Mann–Whitney non-parametric test

Since TLRs trigger microglial activation, we examined microglial reactivity by Iba1 immunohistochemistry 3 days after CpG injection. Microglial surface area, as an index of glial hypertrophy or activation, was quantified both in contralateral and ipsilateral sides of SN of control and mutant mice following a single unilateral injection of CpG ODN. Microglial cells were more hypertrophied in the CpG ODN injected side compared to contralateral side in both controls (*p* = 0.04) and mutant (*p* = 0.04) mice. There was, however, a further increase in hypertrophied microglia in microglial GR mutant mice compared to controls (*p* = 0.04) (Fig. 4a), which implies that GR expression in microglia restrains TLR9 signaling in microglia.

To examine whether exacerbated neuronal damage observed in GR^LysMCre^ mice was associated with inflammatory mediators, we analyzed GR transcriptional regulation of inflammatory genes. First, the results of inflammatory gene expression by RT-qPCR in SN 24 h after CpG ODN or control-ODN injections in control mice showed that CpG ODN has no effect on *TNF-α* (*p* = 0.9), *iNOS*, *MCP-1*, or *I*κB mRNA levels. However, MyD88 mRNA levels were upregulated (*p* = 0.04) (Fig. 4b). In contrast, *TNF-α* (*p* = 0.03), *iNOS* (*p* = 0.03), *MCP-1* (*p* = 0.02), and *MyD88* (*p* = 0.02) mRNA levels were significantly increased by CpG ODN in GR^LysMcre^ mutant mice (Fig. 4b), indicating that TLR9 signaling is robustly stimulated resulting in strong expression of potent inflammatory genes.

The expression of pro-inflammatory genes was verified in primary control and mutant microglial cultures treated with CpG ODN or CpG ODN plus GR agonist, dexamethasone (Fig. 4c). The increase in *TNF-*α, *iNOS*, and *COX2* by CpG ODN treatment was attenuated by dexamethasone in control but not mutant cultures.

### TLR9 traffic to lysosomes enhanced in GR-depleted microglia

To understand how GR regulates TLR9-induced DN death, in comparison with other TLRs that also elicit inflammatory gene expression, we hypothesized that GR may have a role on upstream processes of TLR9 activation, in particular at the level of TLR9 receptor trafficking or at the level of TLR9 cleavage. TLR9 is normally retained in the endoplasmic reticulum (ER) associated with UNC93B1^35,36^. Upon stimulation with CpG ODN, TLR9 translocates to endolysosomal compartment in UNC93B1-dependent fashion, where it undergoes proteolytic cleavage by pH-dependent proteases, notably cathepsins and asparagine endopeptidase (AEP) to yield an active C-terminal fragment, which is competent for signaling^25,37,38^.

**Fig. 5:**
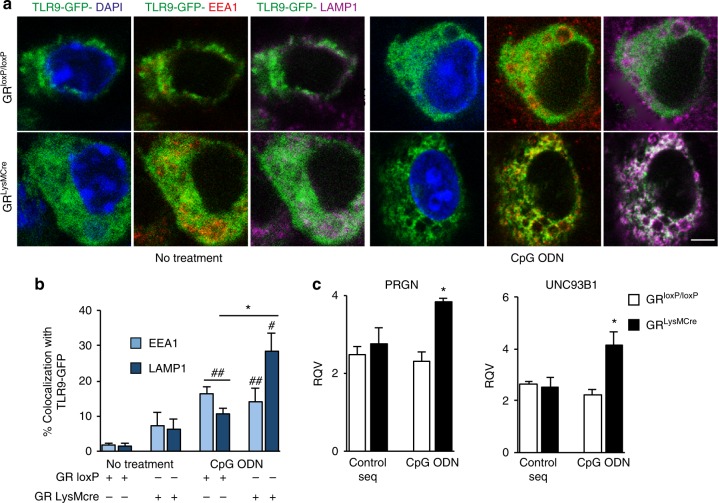
TLR9 translocation to lysosomes is augmented in the absence of microglial GR. **a** Control (C) and mutant (M) primary microglial cells were transfected with TLR9-GFP by nucleofection and 24 h later stimulated or not with CpG-ODN for 1 h. The cells were fixed and stained with specific antibodies for EEA1 (Early Endosome Antigen 1) and LAMP1 (lysosomal marker). Bar = 2 μm. **b** Quantification of TLR9-GFP/EEA1 or LAMP1 co-localization was performed using ImageJ software, from three independent experiments. The results represent the percentage of co-localization of TLR-9 GFP with either EEA1 or LAMP1. *p < 0.05 Control CpG ODN vs. Mutant CpG ODN; # non-treated vs. CpG ODN. **c** RT-qPCR measurement of progranulin (*PRGN*) and *UNC93B1* mRNA levels in SN of control and mutant mice 24 h after control sequence or CpG ODN injection. *p < 0.05 control vs. GR^LysMCre^ mice, *n* = 4–5 per group. Data presented are mean with error bars as s.e.m., Mann–Whtiney test was used statistical significance

To gain insight into the mechanisms underpinning the modulation of TLR9 signaling by GR, we used in vitro primary microglial cultures prepared from cortices of P1 control and mutant pups. The cells were transfected with TLR9-GFP expression plasmid using Amaxa nucleofector system, with transfection efficiency of ≈40%. We analyzed co-localization of TLR9-GFP with early endosomal marker, EEA1 (Early Endosome Antigen 1) and lysosomal marker LAMP1 by confocal microscopy. Results of quantification of co-localized fluorescence signals showed that in the steady state there is little localization of TLR9-GFP with EEA1 or LAMP1-positive compartments both in control and mutant microglia confirming previous reports in dendritic and macrophage cell types (Fig. 5a). Treatment of control and mutant microglial cultures for 1 h with CpG ODN significantly increased TLR9-GFP localization both in early endosomes (EEA1^+^, control cells *p* = 0.004 and mutant cells *p* = 0.02) and lysosomes (LAMP1^+^, control cells *p* = 0.008 and mutant cells *p* = 0.002). The trafficking of TLR9-GFP in LAMP1^+^ compartment was significantly different between microglia from control and mutant pups following treatment with CpG ODN (*p* = 0.001). Indeed, 28% of total TLR9-GFP co-localized with LAMP1^+^ lysosomes in mutant microglia compared to only 14% in control microglia (Fig. 5a).

We also analyzed the expression of *UNC93B1* mRNA whose protein product is involved in TLR9 trafficking and found an increase in the mRNA levels in SN of GR^LysMcre^ mice compared to control GR^loxP/loxP^ mice 24 h after intranigral CpG ODN injection. The expression of progranulin, whose protein product gives rise to granulins, is a co-factor for TLR9^39^, was also elevated in mutant mice (*p* = 0.02 for progranulin and for *UNC93B1*; control vs. mutant mice) (Fig. 5b). Altogether, these results indicate the role of GR in regulating TLR9 translocation to endolysosomal compartment.

### GR controls processing and AEP-dependent TLR9 activation

**Fig. 6:**
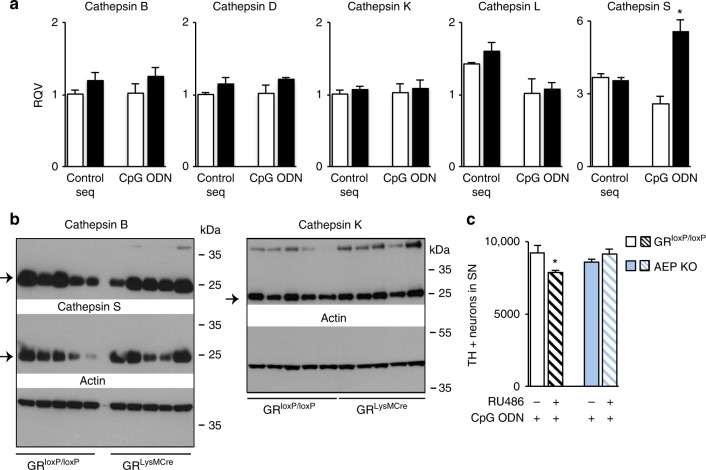
In vivo assessment of the role of microglial GR on TLR9 cleavage enzymes in SN after CpG ODN injection. **a** RT-qPCR analysis of cathepsin B, D, K, L, and S mRNA levels in SN of control GR^loxP/loxP^ and GR^LysMcre^ mutant mice 24 h after intranigral injection of control sequence or CpG ODN. *p < 0.05 control vs. GR^LysMCre^ mice, *n* = 4–5/group. **b** Western blot analysis of cathepsin B, S, and K levels in SN of control GR^loxP/loxP^ and GR^LysMcre^ mutant mice 48 h after CpG ODN injection. The blots were rehybridized with actin antibody. *n* = 5/group. Full gel blots in Supplementary Fig. 4. **c** Quantification of TH-IR neurons in SN of GR^loxP/loxP^ mice and AEP^−/−^ mice, pretreated or not with i.p. injection of GR antagonist RU486, 7 days after a single intranigral injection of CpG ODN. *n* = 5, *p < 0.05; pretreated with RU 486 vs. non- pretreated. All data are presented as mean with error bars as s.e.m. and Mann–Whiney non-parametric test for statistical significance

To examine the involvement of cathepsins and AEP in TLR9 processing in mutant vs. control microglia, we first analyzed, by RT-qPCR, the expression of cathepsins B, D, K, L, and S that can cleave TLR9^40^ and found an increase only for cathepsin S (*p* = 0.02) (Fig. 6a). Western blot analysis of protein levels of cathepsins B, S, and K showed no change in CpG ODN-injected ipsilateral SN of mutant compared to control mice (Fig. 6b). Thus, we checked for the possible involvement of AEP^38^ in GR-mediated regulation of TLR9 activity. We pretreated control and AEP knockout mice with GR inhibitor RU486 (Fig. 1) and then injected CpG ODN in intranigral region. TH + neurons in SN were quantified 7 days after CpG ODN injection in control and AEP^−/−^ mice. The sensitivity to TLR9-induced DN loss observed after RU486 pretreatment in control mice was not observed in mice lacking AEP (Fig. 6c), indicating that cleavage of TLR9 by AEP is an important step in TLR9-induced DN toxicity.

### Lysosomal changes in GR mutant microglia

**Fig. 7:**
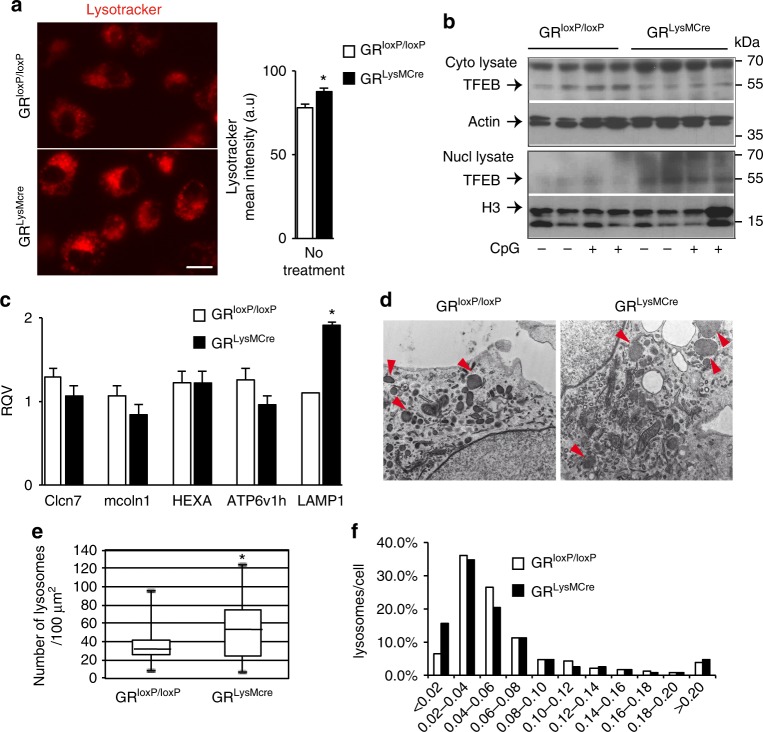
Lysosomal changes in GR^LysMcre^ mutant microglia. **a** Confocal fluorescent images of Lysotracker uptake in living control and mutant primary microglial cells. Bar = 5 μm. The mean fluorescence intensity of Lysotracker was quantified using ImageJ, *n* = 3 separate experiments. *p < 0.05, control vs. mutant cells. **b** A representative western blot experiment shows an increase in nuclear fraction of TFEB in mutant microglial cultures, mirrored by a decrease in the cytoplasmic fraction of TFEB. Full gel blots in Supplementary Fig. 5. **c** In vitro RT-qPCR analysis of expression of lysosomal genes *Clcn7*, *mcoln1*, *HEXA*, *ATP6v1h*, and *lamp1*, in microglial cultures prepared from control and GR^LysMCre^ pups. β-microglobulin gene was used as internal control. *p < 0.05; control vs. GR^LysMCre^ mutant cultures, *n* = 3 independent experiments. The data are presented as mean with error bars as s.e.m. and Mann–Whitney test for statistical significance. **d** Representative EM images of a control and a mutant cell. Red arrows point to lysosomes. **e** Number of lysosomes per 100 μm^2^ in 21 control and 20 mutant cells. Data are expressed as Tukey boxplot where the median is indicated by a horizontal line, the bottom of the box represents the 25th percentile, the top the 75th percentile, whiskers represent minimum and maximum values. **f** Distribution of the lysosome surface (μm^2^). 1386 and 1737 lysosomes were measured, respectively, in control or mutant cells using ImageJ. *χ*^2^ test of independence: *χ*^2^ = 84.81, df 10, *p* < 5.10^–14^

Given that TLR9 cleavage is pH dependent, we analyzed the acidity of lysosomal compartment by treatment of control and mutant microglial cells with Lysotracker, a weakly basic amine fluorescent probe that accumulates in acidic compartments such as lysosomes. The microglial cultures incubated with Lysotracker were imaged and the mean fluorescence intensity of fluorescence-positive lysosomes, an index of the internal pH of the lysosomes, was quantified. The results showed increased mean fluorescence intensity in GR mutant microglial cultures compared to similarly treated control cultures (*p* = 0.003) (Fig. 7a).

Increased Lysotracker staining in GR mutant microglia may be a result of either an overall increase in the lysosomal biogenesis or more acidic lysosomes. TFEB transcription factor is a master regulator of lysosomal biogenesis. We analyzed TFEB levels in the nuclear and cytoplasmic fractions of primary microglial cultures prepared from control and mutant pups and found higher basal levels in the nuclear fraction of GR mutant cultures mirrored by a decrease in the cytoplasmic fraction (Fig. 7b). We also analyzed the expression of *Ccln7*, *mcoln1* (*TRPML1*), *ATP6v1h*, *HEXA*, and *lamp1* genes regulated by TFEB^41^ in control and GR mutant microglial cultures and found an increase in the mRNA level of *lamp1* in the mutant microglial cells compared to controls (*p* = 0.05) (Fig. 7c). To examine whether GR absence in microglia affects lysosome numbers or size, we undertook electron microscopy analyses of lysosomes of control and mutant microglial cells (Fig. 7d). Lysosome numbers and size were quantified in 20 control and mutant cells using ImageJ. Heterogeneity in both numbers of lysosomes ranging from 8 to 120 per 100 μm^2^ and individual lysosomal size ranging from 0.01 to 0. 8 μm^2^ was observed in both control and mutant cells. Nevertheless, as shown in Fig. 7e, mutant cells present a significant increase in the number of lysosomes (median 53 ± 32 lysosomes per 100 μm^2^) compared to control cells (median 32 ± 23 lysosomes per 100 μm^2^). We also analyzed the size of lysosomes in both conditions (Fig. 7f). Comparison of the two distributions with sizes ranging from <0.02 to >0.02 μm^2^ revealed a significant difference in the distribution of the lysosome size (*χ*^2^ = 84.81, df 10, *p* < 5 × 10^–14^). In the mutant cells, there is slight diminution of the number of lysosomes within the 0.02–0.12 μm^2^ range surface, while number of lysosomes with surface <0.02 μm^2^ is increased by 2.4-fold.

### Mitochondrial DNA triggers DN loss through TLR9 activation

**Fig. 8:**
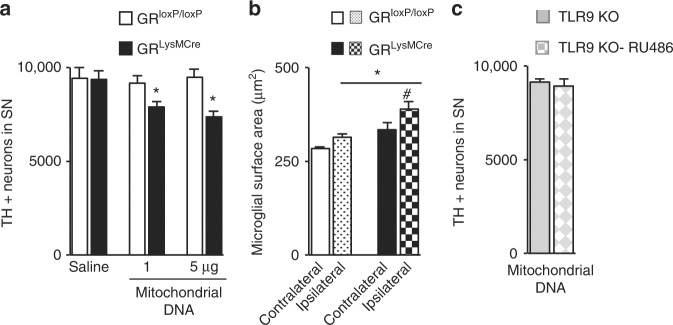
DN loss triggered by mitochondrial DNA in GR^LysMcre^ mutants. **a** TH-IR neurons in SN of GR^loxP/loxP^ mice and GR^LysMCre^ mice quantified 7 days after a single intranigral injection of saline or 1 or 5 μg μl^−1^ mitochondrial DNA. *p < 0.05; GR^loxP/loxP^ mice and GR^LysMCre^ mice. *n* = 5/group. **b** Microglial surface area, an index of microglial hypertrophy, was analyzed by ImageJ in same mice as **a**. The results show increased microglial hypertrophy in GR^LysMCre^ mutant mice compared to controls *p < 0.05 control vs. mutant; #*p* < 0.05 ipsilateral vs. contralateral side. **c** Quantification of TH-IR neurons in SN of TLR9^−/−^ mice, pretreated or not with i.p. injection of GR antagonist RU486, 7 days after a single intranigral injection of 1 μg of mitochondrial DNA. *n* = 5/group. All data are mean with error bars as s.e.m. and Mann–Whitney non-paramtetric test for significance

One of the endogenous ligands reported to activate TLR9 is mitochondrial DNA, which contains CpG DNA repeats, and is released upon cell injury^42^, or which escapes from autophagic degradation^43^. We reasoned that mitochondrial DNA is a plausible endogenous candidate of GR-dependent TLR9 activation in the context of DN neurodegeneration, as there is substantial literature on mitochondrial and autophagic dysfunction in PD pathology^44,45^. Mitochondrial DNA (1 or 5 μg μl^−1^) was injected in the nigral region of control and GR^LysMcre^ mutant mice and TH + DNs in SN were quantified after 7 days. Whereas in the control mice, mitochondrial DNA had no effect, there was DN loss in GR^LysMcre^ mutants (Fig. 8a) (*p* = 0.02; control vs. mutant mitochondrial DNA-injected mice). To test whether mitochondrial DNA injection activates microglia, microglial reactivity was analyzed in SN of mice injected with 1 μg. The results showed increased hypertrophy in ipsilateral side in mutant compared to control mice (*p* = 0.02) (Fig. 8b). To verify that mitochondrial DNA-mediated DN neurodegeneration following GR inhibition is elicited by TLR9, we injected 1 μg mitochondrial DNA in TLR9 knockout mice pretreated or not with RU486. The results of TH-IR DN neuron quantification (Fig. 8c) show that there is no loss of DNs in TLR9 knockout mice pretreated with RU486 (cf. Fig. 1 control mice pretreated with RU486), indicating that DN loss by mitochondrial DNA occurs through TLR9.

## Discussion

We observed a significant reduction in the number of microglia with nuclear GR localization in post-mortem SN of PD suggesting that diminution of GR activity likely promotes DN loss through stimulation of microglial inflammatory signaling cascades. This is corroborated by the results of A53T α-synuclein injection in mice inactivated for GR in myeloid cells or microglia, which resulted in increased degeneration of DNs. Inflammatory signaling cascades initiated by ligation of TLRs are well characterized, as well, microglia are known to express all the members of TLR family. Previously, we reported high TLR9 levels in striatum of post-mortem PD brain tissue^16^. Analysis of TLR9 levels in SN of PD show increased level of C-terminal active fragment of TLR9 protein, suggesting that TLR9 processing may be augmented in SN of PD. These TLR9 protein changes in SN of PD prompted us to examine in mouse models whether TLR9 affects DN survival in SN and elucidate the mechanism(s) through which microglial GR acts to regulate TLR9 inflammatory cascade.

In both GR^LysMcre^ and GR^CX3CR1CreER2^ GR mutant mice, intranigral injection TLR9 ligand, CpG ODN, resulted in significant loss of DNs in SN. This CpG ODN-induced DN loss in GR microglial-macrophage mutants was reversed significantly by systemic administration of TLR9 antagonist COV08-0064, indicating that indeed CpG-ODN activates the TLR9 signaling pathway in SN. Of note, COV08-0064 was reported to specifically antagonize TLR9 activation peripherally; however, its action in brain was not shown. Nevertheless, its physical properties plus our results including that it is not a substrate for Pgp efflux transporter strongly indicates that it can exert effects in brain. To obtain definitive proof of its presence in CNS once injected i.p., synthesis of ^14^C-labeled version of COV08-0064 is now initiated.

The dependency of TLR9 signaling for GR was also corroborated by reducing GR activity with GR antagonist RU486 in control mice, which resulted in DN loss after intranigral CpG ODN injection. The loss of DNs in SN in microglial/macrophage GR mutants was accompanied by forelimb akinesia as revealed by cylinder test.

TLR9 is likely involved in MPTP-triggered experimental Parkinsonism. Thus, we observed a significant reduction of DN loss in TLR9-deficient mice. Our results suggest that this activation is under the control of GR as in the presence of COV08-0064 the increased death of DNs by MPTP was reversed in GR^LysMcre^ mutant mice with no effect in the control mice.

DNs are intrinsically vulnerable to an inflammatory attack^13^, thus, for example, intranigral injection of LPS induces specific loss of DNs through TLR4-innate immune activation of microglia^13^, whereas similar low dose of LPS does not trigger death of striatal neurons^21,46^. In the present study, LPS-activated TLR4 and imiquimod-induced TLR7 activation induced similar DN loss in control and mutant GR^LysMcre^ mice. The results indicating non-significant effect of microglial GR on LPS-induced DN degeneration are intriguing as we previously showed microglial GR control of neuronal death in cortical/striatal region following intra-striatal injection of 1.5 μg LPS as used here^21^. In addition, we also showed that microglial GR regulates inflammatory genes and NF-κB activation induced by LPS. The possibility of high LPS sensitivity in SN compared to striatum and cortex related to greater density of microglia was tested by reducing the dose of LPS to 0.5 and 1 μg. Inter-individual variability in GR^LysMcre^ mice in response to 1 μg LPS was observed suggesting that besides GR other factors control TLR4 signaling in SN microglia.

Morphological analysis of SN microglia in ipsilateral and contralateral side of SN 3 days after CpG ODN injection showed significantly greater hypertrophied microglia in GR^LysMCre^ mutants compared to controls. In line with high microglial reactivity in GR mutants, CpG-ODN also significantly increased mRNA levels of *TNF-α*, *iNOS*, *MCP-1*, *MyD88* with little change in their levels in control mice. This upregulation of pro-inflammatory genes in the absence of microglial/macrophage GR may participate in DN loss.

In examining whether microglial GR affects TLR9 trafficking and cleavage, we uncovered unexpected actions of GR on TLR9 regulation and in microglial functions. The results of TLR9-GFP co-localization in EEA1^+^ early endosomes or in LAMP1^+^ lysosomes in control and microglial GR mutant cultures showed significant expression of TLR9-GFP in lysosomes of mutant microglia after CpG ODN treatment. CpG ODN treatment also led to increased levels of UNC93B1 and progranulin transcripts in mutant GR mice. UNC93B1 is the chaperone protein, required for exit of TLR9 from the ER to the endolysosomes^37^, whereas progranulin, the precursor protein of granulins, was reported as a soluble cofactor for CpG ODN binding to TLR9^25^. Their high expression in mutant GR mice also argues for efficient TLR9 trafficking and processing in endolysosomes.

The major determinant for TLR9 activation is cleavage of its ectodomain near amino acid residue 477 into N-terminal and C-terminal regions by pH-sensitive cathepsin and AEP proteases. This acid-dependent cleavage requires endosome maturation (acidification). Ligand binding to cleaved TLR9 homodimers induces a conformational change that brings the TIR domains into close proximity, enabling MyD88 recruitment^25,37,47^. Our results point to the involvement of AEP in TLR9 cleavage in microglia, as CpG ODN-induced DN loss after pretreatment with RU486 in AEP knock out mice was not observed compared to control mice. As the results point to TLR9 activation in mutant microglial lysosomes, we also examined alterations in microglial lysosomes in the absence of GR. We observed increased Lysotracker fluorescence intensity in GR mutant microglia. Further characterization by transmission EM showed enhanced number and altered size distribution of lysosomes compared to control microglia. Increased level of TFEB transcription factor in nuclear fraction together with augmented expression of LAMP1 in GR mutant microglia imply a change in lysosomal biogenesis and function. Overall, these results show that microglial GR regulates TLR9 trafficking and lysosomal environment.

In our search for relevant TLR9 activators in PD and given that unmethylated CpG sequences are present in mitochondrial DNA, we examined whether mitochondrial DNA can induce dopamine neurodegeneration through TLR9 activation. The role of CpG-rich mitochondrial DNA in triggering inflammation through TLR9 has been previously demonstrated. Oka et al.^43^ showed that mitochondrial DNA that escapes from autophagy-mediated degradation leads to TLR9-mediated inflammatory responses in cardiomyocytes and causes inflammation and heart failure. In systemic lupus erythematous, recognition of self-nucleic acids by TLR7 and TLR9 with ensuing innate inflammation is an important pathological feature, and, interestingly is correlated with reduced GC activity^48^. We found 16–20% DN loss with either 1 or 5 μg mitochondrial DNA which was far less than with CpG ODN. The CpG copy numbers of these two TLR9 ligands are very different. Thus, 1 μg CpG ODN can be estimated to have approximately 4.6 × 10^14^ copies of CpG, whereas similar concentration of mitochondrial DNA has around 1.3 × 10^4^ copies taking into account 850 CpG sites in 16.57 kbp of mitochondrial DNA^49^. This DN loss was GR and TLR9-dependent as it occurred only in GR^LysMCre^ mice and not in TLR9^−/−^ mice treated with RU486. The signals that can activate microglia in SN include oligomeric and fibrillar forms of α-synuclein, neuromelanin, and HMGB1 released from dying DNs^50–53^. Under normal circumstances, self-nucleic acids, which are methylated, are degraded before reaching the endolysosomes where TLR3, TLR7, and 9 are localized. However, if nucleotides are in complexes with HMGB1 released by dying cells, they resist degradation. Recently, high levels of HMGB1 in CSF and serum of PD patients were reported^53^. As well, PD patients harboring G2019S LRRK2 mutation were reported to have a high concentration of mitochondrial DNA in CSF; however, the source of mitochondrial DNA remain unknown^54^. Other possible situations, which may sensitize microglial TLR9 pathway, include CNS and systemic infections as well as brain trauma. Indeed, DNA viruses (known to activate TLR9^55^) such as Herpes simplex virus, Epstein-Barr virus, Cytomegalovirus, and Varicella zoster virus have been associated to Parkinsonism^56^.

**Fig. 9:**
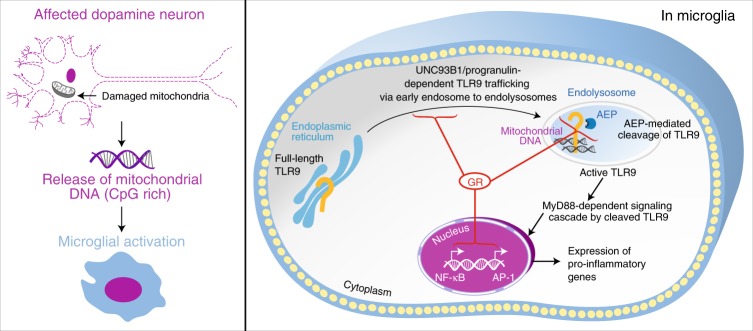
GR regulation of TLR9 activation in microglia that impacts dopamine neuron death. In Parkinson’s disease, chronically high cortisol levels likely compromise GR activity in innate immune cells. TLR9 levels are modulated in post-mortem Parkinson disease brains. Reduced GR activity in microglia permits activation of TLR9 by DAMPS such as CpG-rich mitochondrial DNA released from affected dopamine neurons that selectively exacerbates dopamine neuronal loss in substantia nigra. In microglia, intact GR activity acts as a brake for TLR9 activation and downstream inflammatory gene expression by regulating TLR9 trafficking through expression of UNC93B1 and progranulin and AEP-mediated cleavage of TLR9

Regarding GCs and GR in PD, several studies have reported significantly high circulating levels of cortisol in PD patients^16,57,58^. Chronically high GC levels are known to compromise GR activity in immune cells^59^, which could permit TLR9 induction. Indeed our present work argues for a decreased GR transcriptional activity in microglia. As schematized in Fig. 9, overall, our results suggest that decreased microglial GR activity in SN can lead to an enhancement of dopamine neurodegeneration and pathogenesis in PD patients. Specifically, this loss of GR enables activation of microglial TLR9 inflammatory pathway, which can play a role in the progression of PD pathology.

## Methods

### Human SN autopsy samples

The human post-mortem samples were obtained from brains collected in a Brain Donation Program of the Brain Bank “NeuroCEB” run by a consortium of Patients Associations that include ARSEP (Association for Research on Multiple Sclerosis), CSC (cerebellar ataxias), and France Parkinson. The signed consents were either from the patients or their next of kin in their name, in accordance with the French Bioethical Laws.

SN sections for IF experiments were from three males, one female control subjects: age 75.25 ± 7.4 years, post-mortem delay: 23.87 ± 13.5 h; and from three males, one female PD patients: age 79 ± 2.3 years, post-mortem delay: 30 ± 12.9 h for PD patients. SN western blot samples were from three male control subjects: age 68 ± 10 years, post-mortem delay: 11.5 ± 9.96 h; and two males, one female PD patients: age 78 ± 5.2 years, post-mortem delay: mean 39.5 h for two and one not determined. All values are mean ± s.e.m. Two of the samples in IF and western blot experiments were from same PD patients.

### Mouse models

The *Nr3C1*loxp/loxp;LysMCre mice (thereafter denominated GR^LysMCre^) were generated by crossing *Nr3C1*loxp mice with LysMCre transgenic mice, which express Cre recombinase inserted by homologous recombination under the lysozyme M promoter gene^22,60^. The mice were backcrossed to C57BL/6J for at least 10 generations. Generation of DATicre (Tg BAC-DATiCrefto) and GR^DATiCre^ mice inactivated for GR gene in DNs is described in ref. ^30^. AEP^−/−^ mice were generated in C. Peters’ lab (Freiburg) and backcrossed 11 times on B6 background. TLR9 KO mice were obtained from Drs. N. Doyen and C. Cousin, Pasteur Institute, Paris, and also from Dr. L. Alexopoulou, Centre d’Immunologie de Marseille-Luminy. GR^CX3CR1CreER2^ mice were generated by crossing CX3CR1CreER2 transgenic mice^23^ with *Nr3C1*loxp mice. For Cre recombination in GR^CX3CR1CreER2^ mice, both control and GR^CX3CR1CreER2^ were injected i.p. with 2 mg of tamoxifen (Sigma) per day for 5 days and experiments undertaken 3 weeks after to ensure the disappearance of GR only in CNS microglia^23^.

Mice were group-housed under a controlled photo-period (12-h day–night cycles), at constant room temperature (22 °C) and had access to food and water ad libitum. GR^LysMCre^, GR^DATiCre^, GR^CX3CR1CreER2^ mice were genotyped for the presence of *Nr3C1*loxp allele and Cre or iCre transgene by PCR. All studies were performed in accordance with ethical guidelines of European Convention for the protection of Vertebrate Animals (directives of 2013: 2010/63/EU) and experimental protocol approval from French ministry of Research. Control and mutant littermate mice were randomly grouped for treatment.

### Stereotaxic injections

For stereotaxic surgery, 3–5-months-old GR^DATiCre^, GR^LysMCre^, GR^CX3CR1CreER2^ mutant male mice and GR^loxp/loxp^ control littermates were anesthetized by an i.p. injection of 100 mg kg^−1^ ketamine and 10 mg kg^−1^ xylazine. The scalp was shaved and a small hole was made at the surface for injection into the left substantia nigra using the following stereotaxic coordinates in mouse Paxinos atlas (David Kopf Instruments, Tujunga, CA, USA) from Bregma −2.8 mm anteroposterior, +1.3 mm mediolateral, and –4 mm dorsoventral. In all, 0.5, 1, and 1.5 μg of TLR4 agonist LPS (LPS *Escherichia coli*, serotype 055:B5; Sigma, St. Quentin Fallavier, France), 1 μg of TLR2 agonist (Pam3Cys-Ser- (Lys) 4 Trihydrochloride; Abcam, ab142085), 1 μg of TLR7 agonist, Imiquimod (Enzo Life Sciences, ALX-420-039-M100), 0.96 μg (1 μM) of TLR9 agonist ODN 1826 (TLRgradeTM; 5′**-**TCCATGACpGTTCCTGACpGTT-3′) (Enzo Life Sciences, ALX-746-002-T100) or a control ODN sequence (5′-TCCATGAGCTTCCTGAGCTT-3′) dissolved in PBS were injected using 10 μl Hamilton syringe into the substantia nigra over a 5-min period. For Adeno-associated vector (AAV) serotype 6 PGK-A53T-α-synuclein injections, the stock of 1.97 × 10^13^ gp (genomic particles/ml) was diluted to 2.5 × 10^9^ gp μl^−1^ in saline. 2 μl of AAV-PGK-A53T α-synuclein was injected and mice sacrificed after 8 weeks.

### Intraperitoneal RU486 pretreatment

GR^LysMCre^ mutants, control GR^loxp/loxP^, AEP KO, and TLR9 KO mice were pretreated by an IP injection of 30 mg kg^−1^ RU486 dissolved in 1:9 ethanol:oil 16 h before stereotaxic injection of CpG ODN.

### MPTP and TLR9 antagonist COV08-0064 treatment

Experiments were performed on 3–5-months-old TLR9 k.o. and GR^LysMCre^ mutant mice and their control littermate male mice. For TLR9 k.o. experiment the mice received four i.p. injections of 20 mg kg^−1^ MPTP.HCl or the same volume of saline at 2 h intervals. Mice were sacrificed 7 days after the last injection. For MPTP-COV08-0064 experiment, the GR^loxp/loxP^ and GR^LysMCre^ mutant mice were injected with 120 mg kg^−1^ COV08-0064 (dissolved in saline) 1 h prior to injection of 18 mg kg^−1^ MPTP-HCl. MPTP dose was reduced as increased lethality was observed by combination of COV08-0064-MPTP treatment particularly in GR^LysMCre^ mutant mice during injection paradigm. 24 h after injections, both control and mutant mice recovered in similar manner. For MPTPv+ COV08-0064 group, the mice were injected with the same concentration of COV08-0064 × 1 for 2 consecutive days following MPTP treatment and sacrificed 4 days later.

### Behavior tests

For cylinder test, mice were placed individually inside a glass cylinder (diameter 15 cm, height 30 cm). A camera was placed above the cylinder to allow full vision of the cylinder. The session was videotaped during 5 min for later scoring. No habituation of the mice to the cylinder was made. The assessment was adapted from previous study^32^: the first 20 wall touches with the ipsilateral and the contralateral forelimbs (contacts with fully extended digits), executed independently, were counted. Simultaneous paw touches were excluded from the analysis. Data are expressed as a percentage of contralateral touches, calculated as (contralateral touches)/(ipsilateral touches + contralateral touches) × 100. The analysis performed by the experimenter blinded to the genotype of mice.

For Rotarod test, mice were first habituated to stay on the rotarod apparatus (LSI Letica Scientific Instrument—Rota-Rod LRS) at 5 rpm for 1 day, followed by habituation the next 2 days on the accelerating rotarod with rotarod speed linearly increasing from 5 to 40 r.p.m. over 300 s, so that the latency to fall for all was 3–5 min. One week later the animals were tested on accelerating rotarod three trials/day, at least 10 min interval for 3 days. The latency to fall from the rod was recorded for each trial. Mice remaining on the rod for more than 300 s were removed and their time scored as 300 s. The mice were injected with CpG-ODN the following day and test repeated 1 week later.

### IF and western blot in human brain

Midbrain sections were verified for decrease in TH-IR in PD samples and localization of SN. IF of GR and Iba1 was carried out on 30 μm fresh sections (*n* = 4 for control subjects and 3 for PD patients) and with 10 μm paraffin sections (*n* = 1 PD). As similar results were obtained with sections from fresh-frozen tissues and paraffin-embedded sections, the results were pooled. The method of Monier et al.^61^ was used to achieve Iba1 labeling. Paraffin-embedded sections were deparaffinated in a series of xylene/alcohol solutions, and antigen retrieval was undertaken by heating the sections at 80 °C for 20 min in 10 mM sodium citrate buffer, pH 6.0. Cryostat fresh sections were air-dried before fixation with freshly prepared 4% PFA in PBS solution for 20 min. The sections were rinsed in Tris-buffered saline (TBS) solution containing 0.5% Triton before blocking for 2 h in 2% goat serum in TBS-0.5% Triton. They were incubated with rabbit polyclonal anti-Iba1 antibody (WAKO-1/200) and mouse anti-GR antibody (AbCam-1/200) in 2% goat serum/TBS-0.5% Triton for 48 h at 4 °C. After washes in TBS-0.5% Triton, they were incubated with secondary antibodies: Alexa goat anti-rabbit 488 and donkey anti-mouse Cy3, washed, stained with Hoechst 33342 and mounted using vectashield.

For western blot analysis, SN was dissected and protein homogenates were prepared as reported previously^16^ in lysis buffer containing 62.5 mM Tris pH 6.8, 1% SDS, 10% glycerol, 5% beta-mercaptoethanol, and protease inhibitors (complete cocktail, Roche). 20 μg lysates were loaded onto 10% acrylamide gel. After transfer onto a PVDF membrane, the blots were probed with TLR9 primary antibody (1/500 Imgenex)^16^. The same blot was re-incubated with actin for loading control. After incubation with secondary peroxidase-conjugated antibodies (GE Healthcare) diluted at 1:2000, signals were visualized by using ECL detection kit (GE Healthcare).

### Immunohistochemistry and western blot in mice

For immunohistochemistry, mice were anesthetized with CO_2_ and rapidly perfused transcardially with ice-cold 0.1 M sodium phosphate buffer (PBS) followed by ice-cold 4% paraformaldehyde (PFA) in 0.1 M sodium PBS. Brains were rapidly removed from the skull and post-fixed for 24 h in fresh 4% PFA in PBS solution. 30 μm thick coronal sections were cut serially as described in Ros Bernal et al.^11^ and stored in PBS containing 0.4% sodium azide at 4 °C until use. Sections of substantia nigra, at 180 μm interval, were rinsed in PBS, treated with 0.3% H_2_O_2_/PBS for 15 min and non-specific Fc binding sites were blocked in 2% newborn goat serum (Sigma-Aldrich) in PBS-T (PBS/0.1% Triton X-100); sections were incubated with rabbit polyclonal anti-Iba1 (W1W019–1974, 1:750; Wako Chemicals), or mouse monoclonal anti-TH (MAB 318, 1:1000, Millipore), anti PV25 (1:500, MAB 1572, Sigma) primary antibodies for 24 h (at 4 °C), with constant shaking. After three washes, sections were incubated for 2 h with appropriate anti-mouse or anti-rabbit biotinylated secondary antibodies (1:500; Vector Laboratories). Binding of antibody was detected with avidin-biotin peroxidase ABC kit according to manufacturer’s instructions (Vectastain, Vector Labs) using the chromogen diaminobenzidine (Sigma) as a substrate for the peroxidase. Sections were mounted on superfrost plus slides and dehydrated in graded ethanol series and xylene and then coverslipped. Fluoro-Jade B staining procedure was carried out according to the manufacturer’s instructions (Histo-Chem Inc., Jefferson, AR, USA).

For western blot analysis of cathepsins, protein lysates were prepared in the lysate buffer as described above from punches of ipsilateral SN of control and GR^LysMcre^ mice injected with CpG ODN. 25 μg lysates were loaded on 14% acrylamide gels and after transfer onto a PVDF membrane, the blots were probed at 1/250 with mouse monoclonal anti-cathepsin B (H-5, SC36558 Santa Cruz), rabbit polyclonal anti-cathepsin K (ab85716, AbCam) and mouse monoclonal anti-cathepsin S (E-3 SC271619 Santa Cruz) and actin used as loading control.

### Image analysis

Quantification of DN loss was performed stereologically as described previously^16^ on regularly spaced DAB sections of mesencephalon covering the whole SN (from rostral pole of the SN to the locus coeruleus) by bright-field microscopy using a Nikon microscope (Eclipse, 20× objective) equipped with a semi-automatic stereology system (Mercator software; Explora Nova VisioScan T4.18 system). The genotype of mice was unknown to the investigator at the time of quantification.

Fluoro-Jade B-positive cells were quantified from photomicrographs taken using FITC filter at 4× objective (Nikon). For all quantifications related to activated microglia, DAB-labeled sections were used. ImageJ software (NIH, USA) was used for quantification of Fluoro- Jade B-positive neurons, microglial densities, as well as microglial surface area.

For human brain sections, stacks of consecutive images at 2 μm intervals were acquired sequentially using 40× objective on a confocal laser-scanning Leica microscope. Three to seven fields were selected at random in SN per section then reconstructed and quantified using ImageJ software.

### RT-quantitative PCR

Mice were euthanized 24 h after CpG injection and their brains snap frozen in isopentane at −25 °C. The ipsilateral lesioned and contralateral non-lesioned SN regions were rapidly dissected by punch at −5 to −10 °C. Total RNA was prepared using the RNeasy lipid mini kit (Qiagen) in sterile conditions. The RNA integrity and concentration was determined by using Nanodrop.

In all, 1 μg of total RNA from whole tissue and 500 ng from cell cultures were used for cDNA synthesis with Superscript III (Invitrogen, St. Aubin, France). HPRT and β-microglobulin were used as internal controls for whole tissues and in vitro microglial cultures, respectively. qPCR experiments were carried out on Roche Light Cycler 480-II (Roche Diagnostic) using Syber Green master mix.

### In vitro experiments on primary microglial cells from P1 control and GR^LysMCre^ pups

Cerebral hemispheres were dissected and cortices extracted from newborn mice after removal of meninges. After dissociation and homogenization, cells were seeded at 30,000 cells ml^−1^ in DMEM containing 10% heat-inactivated FCS on poly-ornithine plated dishes. Medium was changed at days 2 and 4, and microglial cells were collected at day 12 by shaking culture dishes to detach cells adhering to the astrocyte monolayer, as described.

Freshly collected microglia were seeded on 24-well plates in DMEM containing 4% FCS. They were treated 48 h later with, 1 μm CpG ODN for 1 h, in DMEM medium containing 0.5% FCS. Microglial cells were collected in RLT buffer from RNeasy Mini Kit (Qiagen) and stored at −80 °C for total RNA extraction.

### Transfection with TLR9-GFP plasmid

10^6^ microglial cells harvested were transfected with 1 μg of cDNA coding for TLR9-GFP^38^ using Amaxa Mouse macrophage nucleofector kit (Lonza), according to the manufacturer’s instructions. 3 h later, when the cells had adhered, the medium was changed to: DMEM containing 4% FCS, 2 mM glutamine (Gibco 25030-024), 50 μm 2mercaptoethanol, GM-CSF 0.1 μg ml^−1^ (R&D Research 415). 24 h later, cells were stimulated with TLR9 agonist, CpG ODN.

### IF of microglia in vitro

Autofluorescence was quenched with ammonium chloride/PBS. Non-specific Fc binding sites were blocked with 0.25% gelatin in PBS. Primary antibodies against EEA1 (610457 Brand BD Transduction Laboratories) and LAMP1 (553792BD pharmingen) were incubated overnight at 4 °C. Secondary antibodies coupled to cy3, Alexa 488, cy5 (Invitrogen) were used at 1/400 dilution for 2 h followed by 15 min in a PBS solution containing 4′,6′-diamidino-2-phenylindole (DAPI) (1/2000) and then mounted for examination under a fluorescence microscope.

### Quantification of co-localization

Stacks of consecutive images at 0.15 μm intervals were acquired sequentially with three lasers at 488 nm (TLR9-GFP) and 555 nm (EEA1) and 633 nm (LAMP1) at objective (63×) on a confocal laser-scanning Leica microscope. Confocal fluorescent images were converted to binary images in ImageJ. The fluorescence across one entire image was determined; a threshold for positive staining was set for each channel (green, red, or far red). The percentage of co-localization between two given channels was derived from the number of positive pixels common to these two channels (TLR9 GFP and LAMP1; or TLR9 GFP and EEA1, for example), divided by the number of positive pixels within the TLR9-GFP image.

### Lysotracker treatment and image acquisition

Microglial cells were plated in 8-well ibi-treated (IBIDI—optimal cell adhesion) chamber followed by treatment with 60 nM lysotracker red (DND 99 Invitrogen) in phenol-free 0.5% FCS DMEM for 30 min, in a thermostated chamber at 37 °C and CO_2_. Images of living cells were taken with the confocal microscope 63× objective using the 577 nm filter.

### Nuclear and cytoplasmic fractionation and immunoblots

Cells were trypsinized at 37 °C for 5–10 min. Reaction was stopped by adding DMEM containing 10% FCS, then harvested and centrifuged at 1300 rpm for 20 min at 4 °C. Pellets were then resuspended in 100 μl Buffer A (10 mM HEPES-KOH pH 7.9, 1.5 mM MgCl2, 10 mM KCl, 0.5 mM DTT, 0.2 mM PMSF with protease inhibitors), allowed to swell on ice for 10 min, vortexed then centrifuged for 10 s. Supernatant containing cytoplasmic proteins was recuperated and stored at −80 °C. Pellets were resuspended in 100 μl RIPA buffer (150 mM KCl, 50 mM HEPES, 0.5% NP40, 0.2% Na deoxycholate, 0.5 mM EGTA, 0.5 mM EDTA, with protease inhibitors), centrifuged at 12,000 rpm for 15 min at 4 °C. Supernatant containing nuclear proteins was recuperated and stored at −80 °C. 20 μg of protein samples were loaded on Novex NuPage 4–12% Bis-Tris gradient gels (Invitrogen). After transfer onto a PVDF membrane, the blots were probed with TFEB primary antibody (SAB4503154, Sigma). The same blots were re-incubated with either actin or histone H3 antibodies for loading control. After incubation with secondary peroxidase-conjugated antibodies (GE Healthcare) diluted at 1:2000, signals were visualized by using ECL detection kit (GE Healthcare).

### Transmission electron microscopy

Primary microglial cells were fixed in 2% glutaraldehyde (Agar) diluted in 0.1 M PBS at pH 7.4. Cells were washed and incubated for 1 h in 1% osmium tetroxide (EMS) reduced with 1.5% potassium ferrocyanide diluted in 0.1 M sodium cacodylate (EMS). Cells were dehydrated through graded concentration of ethanol (50-70-96-100%) and infiltrated in epoxy resin (Agar 100 resin kit, Agar) at room temperature according to the manufacturer’s instructions and polymerized for 48 h at 60 °C. Ultrathin sections (70 nm) were cut with an ultramicrotome (Ultracut, Leica Microsystems) and collected on 200 Mesh copper grids (Agar). Sections were stained with 2.5% aqueous uranyl acetate (Prolabo) and 1% lead citrate (Agar) before observation at 80 kV with a Zeiss 912 Omega TEM, equipped with side-mounted 2k × 2k Veleta (Olympus) CCD camera controlled with iTEM (Olympus) software. Images were analyzed using ImageJ. The number and surface of individual lysosomes per μm^2^ were calculated in 21 control cells (1386 lysosomes) and 20 mutant cells (1787 lysosomes). After counting, distributions of lysosome surface were compared with the *χ*^2^ test of independence, according to the formula in Eq. (1), where *A*_*ij*_ and *E*_*ij*_ refers, respectively, to the observed and the theoretical values:

1 $$X^2{\mathrm{ = }}{\sum} {{\sum} {\frac{{\left( {\left( {A_{ij}} \right) - E_{ij}} \right)^2}}{{E_{ij}}}} }.$$

### Mitochondrial DNA preparation

Mitochondria were isolated from the brains and livers of C57Bl6 mice using “mitochondria isolation kit for tissue” (Thermo Scientific), according to the manufacturer’s protocol. Mitochondrial DNA was extracted from the mitochondrial pellet according to the procedure described by Palva and Palva^62^. Briefly, mitochondrial pellet was resuspended in 200 μl buffer containing 50 mM glucose, 10 mM EDTA, and 25 mM Tris-HCl (pH 8) on ice. To lyse the mitochondria and denature the contaminating nuclear DNA, 400 μl alkaline SDS solution (0.2 M NaOH, 1% SDS) was added and the suspension thoroughly mixed. After 5 min incubation on ice, 300 μl of 3 M potassium acetate was added, and the contents of the tube gently mixed. The tube was maintained at −70 °C for 2 min and centrifuged for 10 min at 10,000 × *g*. 750 μl of the clear supernatant was removed and the mtDNA precipitated by adding 450 μl isopropanol and holding the tube for 5 min at −70 °C.

The precipitate was collected by centrifugation, washed with 70% ethanol and and the pellet stored at −20 °C. The mtDNA pellet was resuspended in 100 μl of 10 mM Tris-HCl (pH 8) and 1 mM EDTA, treated with DNase-free RNase, and phenol extracted. DNA was re-precipitated with ethanol in the presence of 150 mM NaCl overnight at −80 °C. The precipitate was washed with 70% ethanol, dried, and resuspended in 15 μl of 10 mM Tris-Cl (pH 8) and 1 mM EDTA.

### Statistical analysis

Data are expressed as mean ± s.e.m. Statistical analysis was performed using Mann–Whitney tests unless otherwise stated. For animal and human studies, *n* = 5 for most data points, or 3–4 as stated. Differences of *p* < 0.05 were considered statistically significant. Statistical analyses were carried out using StatView 5.0 software.

### Data availability

The data supporting the finding in this study are available from corresponding author upon request.

## Electronic supplementary material

Supplementary Information
