# Author Correction: TLR9 activation via microglial glucocorticoid receptors contributes to degeneration of midbrain dopamine neurons

**DOI:** 10.1038/s41467-018-05680-w

**Published:** 2018-08-01

**Authors:** Layal Maatouk, Anne-Claire Compagnion, Maria-Angeles Carrillo-de Sauvage, Alexis-Pierre Bemelmans, Sabrina Leclere-Turbant, Vincent Cirotteau, Mira Tohme, Allen Beke, Michaël Trichet, Virginie Bazin, Bobby N. Trawick, Richard M. Ransohoff, François Tronche, Bénédicte Manoury, Sheela Vyas

**Affiliations:** 1 Institute of Biology Paris Seine, Gene Regulation and Adaptive Behaviors Team, Department of Neuroscience Paris Seine, Sorbonne Université, CNRS UMR 8246 & INSERM U1130, 9 Quai Saint Bernard, F-75005 Paris, France; 2 CEA, DRF, Institut François Jacob, Neurodegenerative Diseases Laboratory, Molecular Imaging Research Center (MIRCen), CNRS, CEA, Université Paris-Sud, Université Paris-Saclay (UMR9199), F-92265 Fontenay-aux-Roses, France; 3 IHU-A-ICM–Neuro-CEB, Plateforme de Ressources Biologiques (PRB), Hôpital de la Pitié-Salpétrière, 47 Boulevard de l’Hôpital, F-75013 Paris, France; 4 INEM, INSERM U1151-CNRS UMR 8253, Hôpital Necker, Université Paris Descartes, Sorbonne Paris Cité, Faculté de Médecine, 149 Rue de Sèvres, F-75005 Paris, France; 5 Institute of Biology Paris Seine, Electron Microscopy Facility, Sorbonne Université FR3631, 9 Quai Saint Bernard, F-75005 Paris, France; 6 Center for Organic Chemistry, Mallinckrodt Pharmaceuticals, 3600N. Second Street, B81-T l, 63147 St. Louis, MO USA; 7 Third Rock Ventures, 02116 Boston, MA USA

Correction to: *Nature Communications* ; 10.1038/s41467-018-04569-y; published online 22 June 2018

The originally published version of this Article contained an error in the subheading “Microglial GR does not affect DN loss triggered by TLR4 and TLR7”, which was incorrectly given as “Microglial GR does affect DN loss triggered by TLR2 and TLR4.” This has now been corrected in both the PDF and HTML versions of the Article.

