# Supplementary material for: TLR9 activation via microglial glucocorticoid receptors contributes to degeneration of midbrain dopamine neurons
Source: Nat Commun. 2018 Jun 22;9:2450. doi: 10.1038/s41467-018-04569-y (PMC6015079; doi:10.1038/s41467-018-04569-y)
Supplement: Supplementary file 1 — Supplementary Information [file 41467_2018_4569_MOESM1_ESM.pdf]

Supplementary Fig.1

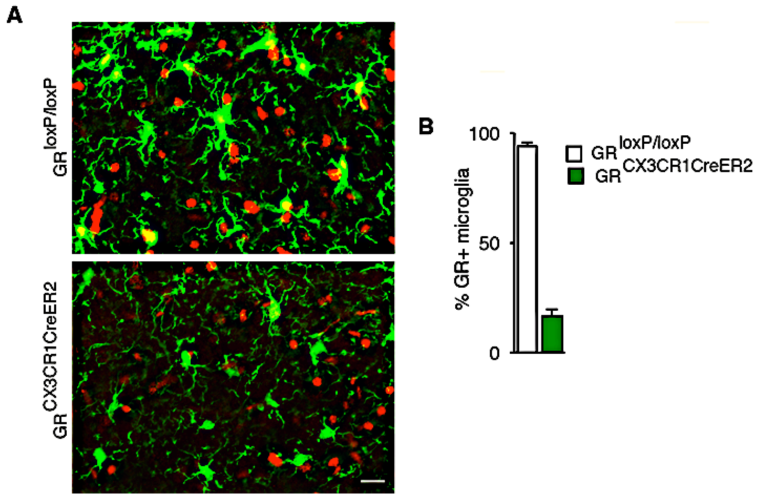

Verification of GR absence in SN microglia in GR ^{CX3CR1CreER2} mice

(**A**) Expression of GR (*red*) in Iba 1 positive microglia (*green*) in SN of control GR $^{loxP/loxP}$ and mutant GR ^{CX3CR1CreER2} mice. Bar=10μm (**B**) Microglia expressing or not GR were quantified in control and GR ^{CX3CR1CreER2} mice. n=4/group. Data are mean with error bars as s.e.m.

Supplementary Fig.2

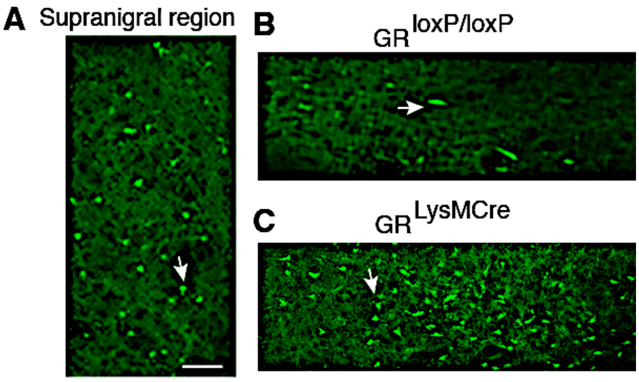

Confocal images of Fluoro-Jade B labeling neuronal degeneration after CpG ODN injection

Representative images of degenerating neurons visualized by Fluoro-Jade B staining in supranigral region (**A**), in SN of control (**B**) and $GR^{LysMcre}$ mice (**C**) mice after CpG ODN injection. Bar= 50 μm

Supplementary Fig.3

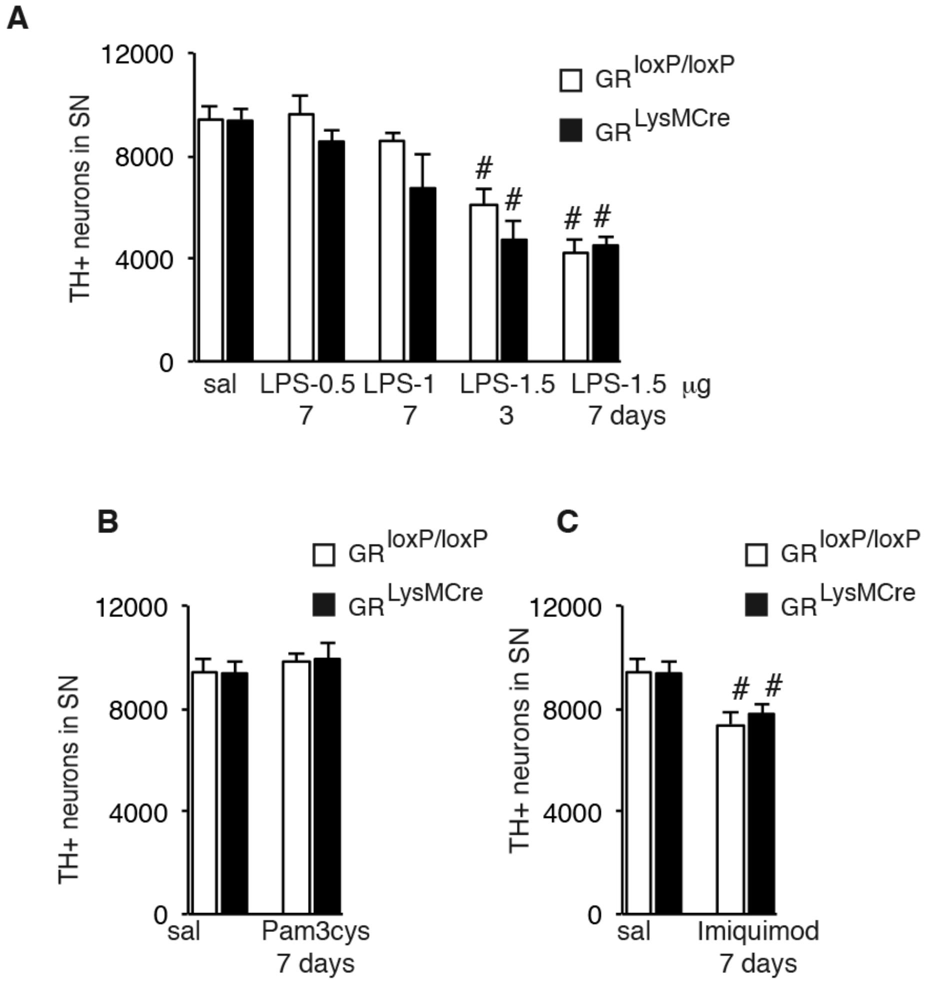

The role of micorglial/macrophagic GR in regulating TLR4, TLR2 and TLR7 mediated loss of DNs

TH-IR neurons in SN were quantified in $GR^{LysMCre}$ and $GR^{loxP/loxP}$ mice 3 or 7 days (as indicated) after a single intranigral injection of either saline (sal), different concentrations of LPS (TLR4 ligand), pam3cys (TLR2 ligand), imiquimod (TLR 7 ligand); # $p < 0.05$ LPS or imiquimod saline vs n=5/group. Data are presented as mean and error bars as s.e.m, with Mann Whitney test for statistical significance.

Supplementary Fig.4

Uncropped immunoblot images

Fig. 2A

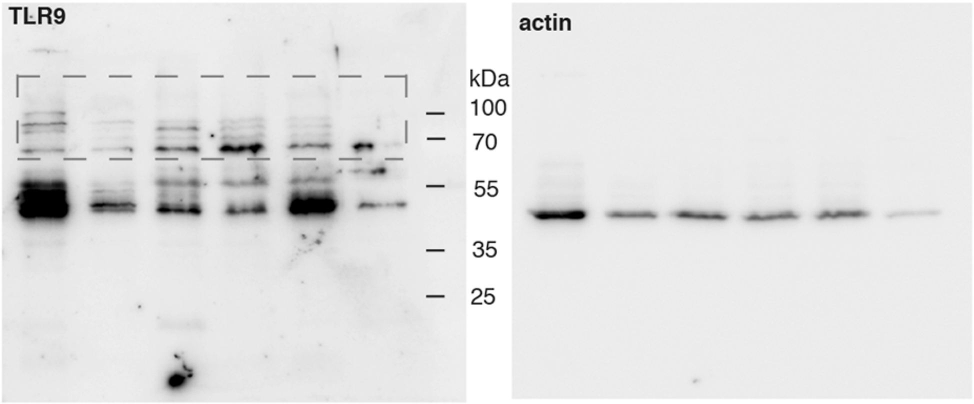

Fig. 6B

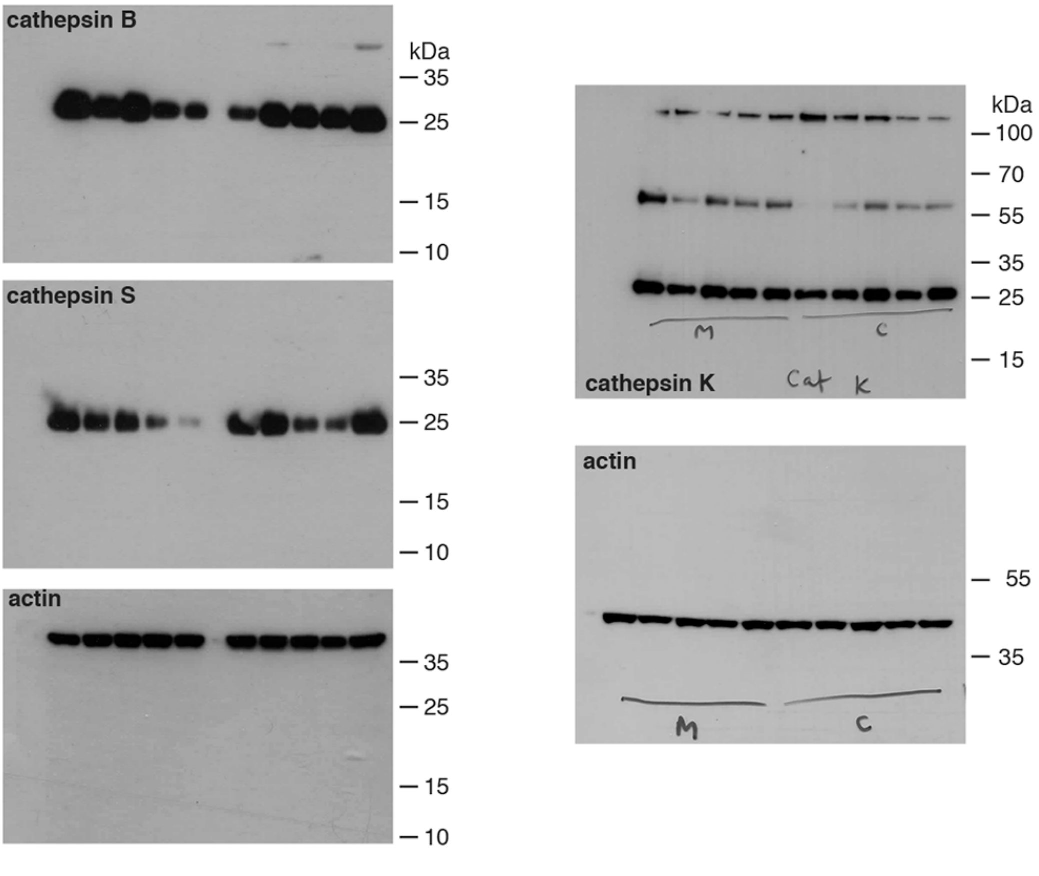

Uncropped western blot gel images of cathepsin B, S and K with actin as loading control. Cropped gel images (around red broken lines) are shown in Fig.2A and Fig. 6B.

Supplementary Fig.5

Uncropped immunoblot images

Fig. 7B

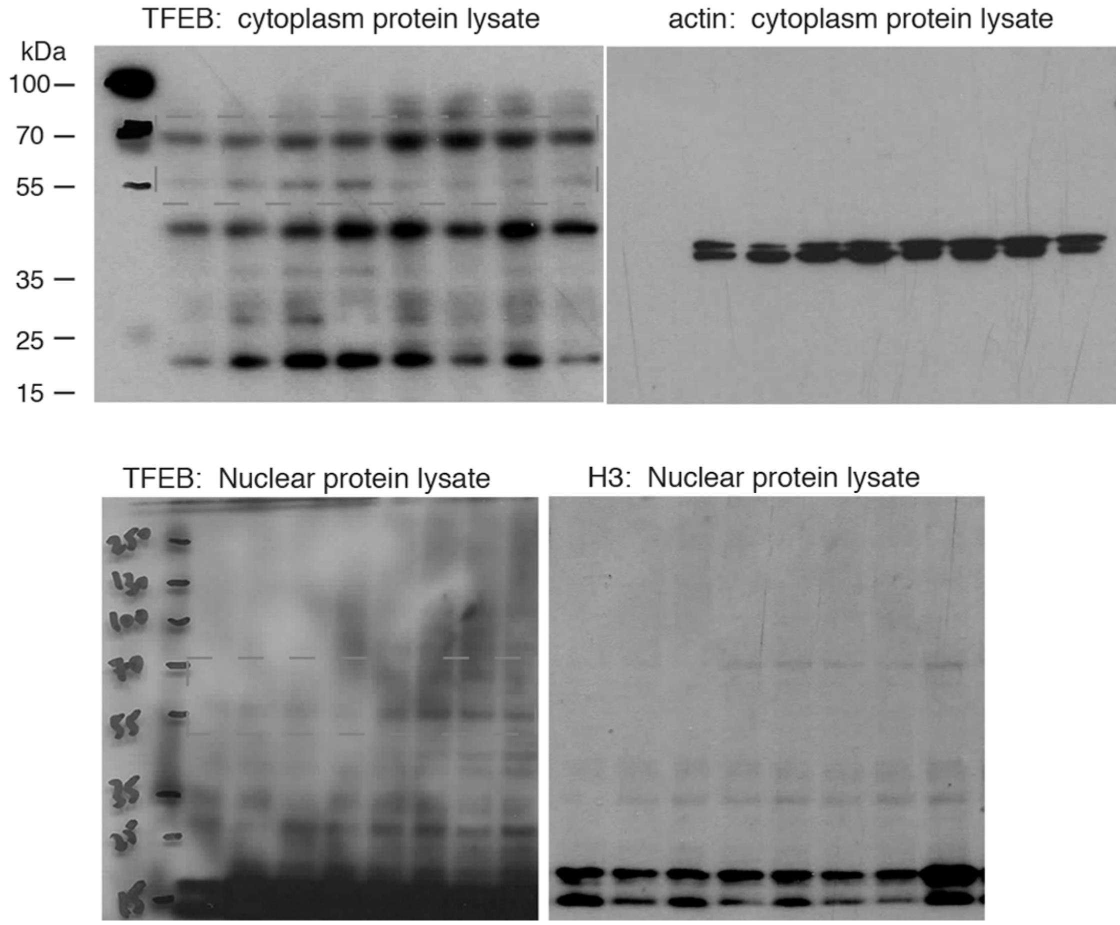

Full western blot gel images of cytoplasmic and nuclear TFEB and actin or histone H3 as loading control, respectively, in microglial primary cultures from $GR^{loxP/loxP}$ and $GR^{LysMCre}$ mice. Cropped images (around red broken lines) are shown in Fig. 7B.

Supplementary note 1

COV08-0064 is a novel small molecule, heterocyclic compound with unique structural and functional characteristics. While its octahydrophenanthrene-based structure resembles that of the naturally derived (-)-morphinan class of compounds such as (-)-morphine, (-)-codeine and (-)-naltrexone, COV08-0064 is structurally distinct from these compounds because it possesses the opposite stereochemistry at each chiral center within the core morphinan scaffold. COV08-0064 is therefore classified as a (+)-morphinan compound. The enantiomeric stereochemical configuration of the COV08-0064 structural core provides it with the drug-like properties that are typical of (-)-morphinans but with functional properties that differentiate it from that class of compounds. For example, unlike (-)-morphinan compounds, COV08-0064 has been shown to lack any functional agonist or antagonist activity at opioid receptors. It therefore lacks any of the negative side effects traditionally associated with opioid receptor activation.

Hoque et al (2014) [1] showed that COV08-0064 has a high specificity for TLR9 but not other TLRs, in contrast to DNA-based TLR9 antagonists, which can also inhibit TLR7 and exert immunoregulatory actions independent of TLR9. Moreover, in contrast to the inherently poor oral bioavailability of the high molecular weight, polyanionic DNA based TLR9 antagonists; COV08-0064 had 55% oral bioavailability and half-life of 149 minutes. The concentration of 80mg/kg COV08-0064 (as used in this study) was protective against ischemia/reperfusion liver injury and TLR9 cleavage and down-stream signaling cascade were inhibited [2].

Although no information has been reported about the ability of COV08-0064 to enter CNS, there are several points that strongly indicate that COV08-0064 exerts its inhibitory actions also in CNS:

1/ Based on physicochemical and structural attributes, it can be predicted with confidence whether a compound can cross blood brain barrier (BBB) by passive diffusion. Lipinski's "rule of five" relates BBB permeability to molecular weight, lipophilicity, polar surface area (PSA), hydrogen bonding and charge. Lipinski's conclusion is supported by experimental and computational Quantitative Activity Relationship (QSAR) approaches [3,4]. As shown in **Table1** our calculated values of "five rules" fall well within the range of predicted values [3-5] for permeability through BBB:

Supplementary table 1

Properties	Value calculated for COV08-0064	Predicted optimum values for traversing BBB
Molecular weight	356,47 Da	<400Da
LogP	1.95	1.5-2.5
TPSA	64.95	60-70
Hydrogen bonding capacity:	3	≤3
Hydrogen bond acceptor	5	≤7

2/ The activity of Pgp efflux transporter in the calcein acetoxymethyl ester fluorescence assay (Calcein-AM) using the MDR1-MDCKII cell line is an early predictive assay for screening of compounds to penetrate the BBB. Our data in Table 2 clearly shows that COV-08-0064 is not a substrate for the Pgp efflux transporter, as there was negligible inhibition at any of the doses studied.

Supplementary table 2

Concentration of COV08-0064 (µM)	Average % Inhibition in Calcein AM Fluorescence Assay
1	-0.65
30	-0.4
100	-0.25

References

1 Hoque, R. *et al.* A novel small-molecule enantiomeric analogue of traditional (-)-morphinans has specific TLR9 antagonist properties and reduces sterile inflammation-induced organ damage. *J Immunol* **190**, 4297-4304, doi:10.4049/jimmunol.1202184 (2013).

2 Shaker, M. E., Trawick, B. N. & Mehal, W. Z. The novel TLR9 antagonist COV08-0064 protects from ischemia/reperfusion injury in non-steatotic and steatotic mice livers. *Biochem Pharmacol* **112**, 90-101, doi:10.1016/j.bcp.2016.05.003 (2016).

3 Pajouhesh, H. & Lenz, G. R. Medicinal chemical properties of successful central nervous system drugs. *NeuroRx* **2**, 541-553, doi:10.1602/neurorx.2.4.541 (2005).

4 Mikitsh, J. L. & Chacko, A. M. Pathways for small molecule delivery to the central nervous system across the blood-brain barrier. *Perspect Medicin Chem* **6**, 11-24, doi:10.4137/PMC.S13384 (2014).

5 Carpenter, T. S. *et al.* A method to predict blood-brain barrier permeability of drug-like compounds using molecular dynamics simulations. *Biophys J* **107**, 630-641, doi:10.1016/j.bpj.2014.06.024 (2014).
